# Isolated psychosis during exposure to very high and extreme altitude – characterisation of a new medical entity

**DOI:** 10.1017/S0033291717003397

**Published:** 2017-12-05

**Authors:** Katharina Hüfner, Hermann Brugger, Eva Kuster, Franziska Dünsser, Agnieszka E. Stawinoga, Rachel Turner, Iztok Tomazin, Barbara Sperner-Unterweger

**Affiliations:** 1 Department of Psychiatry, Psychotherapy and Psychosomatics, University Hospital of Psychiatry II, Medical University Innsbruck, Innsbruck, Austria; 2 Institute of Mountain Emergency Medicine, EURAC research, Bolzano, Italy; 3 Medical University Innsbruck, Innsbruck, Austria; 4 Management and Committees, EURAC research, Bolzano, Italy; 5 Department of Family Medicine, Faculty of Medicine, University of Ljubljana, Slovenia

**Keywords:** Acute mountain sickness, altitude, high altitude cerebral oedema, psychosis, schizophrenia

## Abstract

**Background:**

Psychotic episodes during exposure to very high or extreme altitude have been frequently reported in mountain literature, but not systematically analysed and acknowledged as a distinct clinical entity.

**Methods:**

Episodes reported above 3500 m altitude with possible psychosis were collected from the lay literature and provide the basis for this observational study. Dimensional criteria of the Diagnostic and Statistical Manual of Mental Disorders were used for psychosis, and the Lake Louise Scoring criteria for acute mountain sickness and high-altitude cerebral oedema (HACE). Eighty-three of the episodes collected underwent a cluster analysis to identify similar groups. Ratings were done by two independent, trained researchers (*κ* values 0.6–1).

**Findings:**

Cluster 1 included 51% (42/83) episodes without psychosis; cluster 2 22% (18/83) cases with psychosis, plus symptoms of HACE or mental status change from other origins; and cluster 3 28% (23/83) episodes with isolated psychosis. Possible risk factors of psychosis and associated somatic symptoms were analysed between the three clusters and revealed differences regarding the factors ‘starvation’ (χ^2^ test, *p* = 0.002), ‘frostbite’ (*p* = 0.024) and ‘supplemental oxygen’ (*p* = 0.046). Episodes with psychosis were reversible but associated with near accidents and accidents (*p* = 0.007, odds ratio 4.44).

**Conclusions:**

Episodes of psychosis during exposure to high altitude are frequently reported, but have not been specifically examined or assigned to medical diagnoses. In addition to the risk of suffering from somatic mountain illnesses, climbers and workers at high altitude should be aware of the potential occurrence of psychotic episodes, the associated risks and respective coping strategies.

## Introduction

‘*I first met Jimmy on the Balcony, a cold windswept snow shelf high up on the southeast ridge of Mount Everest. At an altitude of more than 8200 meters our introduction had been brief, with little more than a muffled “hello” and a few words of encouragement passing between us. Over my right shoulder, obscured by the bulky oxygen mask and the rim of down that smothered my face, I was sure I could see Jimmy moving lightly in the darkness. But despite him remaining close by me for the rest of the day, I didn't see him again*’ (Windsor, 2008).

A wealth of personal accounts are available in non-medical literature relating to mountaineering, mainly describing symptoms of psychosis while exposed to very high (3500–5500 m) and extreme (>5500 m) altitude. Psychosis has been proposed to benefit climbers where hallucinations consisted of voices or people encouraging them towards survival behaviour, however, it can also prove detrimental, resulting in potential misjudgements of dangerous scenarios. The dimensional assessment of psychosis in the Diagnostic and Statistical Manual of Mental Disorders, Fifth Edition (DSM-5) (American Psychiatric Association, 2013) includes the following core symptoms: hallucinations, delusions, disorganised speech, abnormal psychomotor behaviour and negative symptoms; and additionally impaired cognition, depression and mania (American Psychiatric Association, 2013; Barch *et al.* 2013). Psychosis is a hallmark of schizophrenia, but can also occur in mood or substance abuse disorders, or as part of the syndrome of organic brain dysfunction (classified as delirium in DSM-5) (American Psychiatric Association, 2013).

The professional medical knowledge and climbers’ awareness of somatic high-altitude (HA)-related symptoms has increased over the past decades, but research on HA psychosis is still scarce. Available reports currently only consist of the analyses of hallucinations or delirium in individual cases or case series (Ryn, 1988; Brugger *et al.* 1999; Basnyat, 2002), plus the description of hallucinations in the context of HA cerebral oedema (HACE) (Wu *et al.* 2006).

By many authors, HACE is considered an extension of acute mountain sickness (AMS) (Hackett & Roach, 2004), but it is now proposed that the continuum of AMS-HACE comprises more than one pathophysiological process (Hall *et al.* 2014). HACE is diagnosed in persons with AMS who also demonstrate ataxia or mental status changes, such as lethargy/lassitude, which can progress to delirium, coma and death (Hackett & Roach, 2004). The reported incidence of HACE varies significantly, probably due to differences in diagnostic criteria and assessment methods as well as limited epidemiological research (Hackett & Roach, 2004). HACE is very rarely reported below 4000 m and the lowest reported altitude of incidence is 2100 m (Hackett & Roach, 2004). Most studies report incidences of 0.1–2.0% at altitudes between 3500 and 5500 m (Hochstrasser *et al.* 1986; Hackett *et al.* 1998; Wu *et al.* 2006), but in situations of fast ascent numbers as high as 31% have been found (Basnyat *et al.* 2000). In the absence of appropriate treatment or rapid descent HACE often leads to death within 24 h (Willmann *et al.* 2014). By contrast, those people who describe symptoms of psychosis in personal accounts in the available lay literature related to mountaineering, have returned from the mountain safely without professional rescue and lacking neurological sequelae.

The primary aim of this study was to assess the frequency of HA psychosis and its association with AMS and HACE, or mental status change from other origins. The primary hypothesis was that isolated psychotic episodes may occur at HA without signs and symptoms of AMS or HACE and therefore can be defined as an independent HA-related pathology. Secondarily, the potential risk factors and somatic comorbidities of HA psychosis and its potential association with near accidents or accidents were evaluated.

## Methods

### Included cases

The basis for inclusion was a survey of the lay literature relating to mountaineering performed via a search on ‘Amazon.de’ (accessed on 25 July 2014) using the German keyword ‘Bergsteigen’ (mountaineering) and the filter ‘Biographien und Erinnerungen’ (biography and history). The results were sorted by relevance and the first 100 hits were analysed. Additionally, the authors of the study contributed books from their private collections for analyses (Supplemental Material 1). Episodes were identified and extracted from the literature via the following criteria: (1) the minimum altitude of 3500 m was reached; (2a) a detailed description of the episode with at least three somatic or psychiatric criteria (regardless if pathological or normal) were present; or (2b) a significant change in the course of the tour occurred that was not attributable to external circumstances (i.e. turning back, change of target, accident or near accident).

### Qualitative data analysis

One hundred and two identified episodes were digitised, numbered and imported in to MAXQDA^®^ (Verbi GmbH, Berlin, Germany). Codes for each symptom or factor were defined and marked in the text. Data from two independent, trained raters were then exported into Microsoft Exel^®^. In cases of divergent ratings, a third rater was involved. The inter-rater reliability was calculated (Cohen's *κ*) using SPSS^®^ software. Inter-rater *κ* was substantial (0.60–0.79) to outstanding (>0.80) for all rated symptoms or factors (Landis & Koch, 1977).

### Demographic and tour-related information

Demographic factors and tour-related information were recorded: name of the mountain/mountain range; altitude at which episode occurred (in meters); altitude gain on the day before the symptoms occurred [⩽400 m altitude gain, >400 m altitude gain, not available (NA): factor ‘ascent’]; duration of episodes (shorter than 1, 1–24, 1–7 days, >1 week, NA: factor ‘duration’); whether the subject was alone (YES/NO/NA: factor ‘alone’) or in a situation of acute danger (YES/NO/NA: factor ‘danger’) when the symptoms occurred, or if the episode was self-reported or observed (YES/NO/NA: factor ‘self-report’).

### Scoring of psychiatric symptoms

DSM-5 dimensional criteria (as reflected in the Clinician-Rated Dimensions of Psychosis Symptom Severity Scale, CRDPSS) were used for assessment of psychosis (American Psychiatric Association, 2013; Barch *et al.* 2013). The CRDPSS includes the following symptoms: hallucinations, delusions, disorganised speech, abnormal psychomotor behaviour, negative symptoms, impaired cognition, depression and mania. Each symptom was assessed as either present (YES) or absent (NO), or alternatively if no information was available on this specific symptom (NA). According to DSM-5, at least two criteria had to be present for the diagnosis of psychosis, with one of them being delusions, hallucinations or disorganised speech (primary symptoms of psychosis) (American Psychiatric Association, 2013).

Additionally, the following sub-categories were rated as follows: (i) for hallucinations the modality (visual, acoustic, somaesthetic, olfactoric, gustatory, third person phenomenon), plus the individual's personal connotation of the episode (positive, neutral, negative); (ii) for abnormal psychomotor behaviour, whether there was a decrease or increase; and (iii) for negative symptoms, whether there was a decrease in motivation, communication or affective expression. Since the whole spectrum of cognitive abilities cannot be extracted from a written description, formal thought disorders (slowed, accelerated, automated or disorganised), which have been shown to correlate with cognition in psychosis, were also assessed (Subotnik *et al.* 2006). Anxiety was added as an additional point. Illusions and depersonalisation were grouped together with hallucinations as perceptional disturbances.

### Associated somatic conditions and diagnosis of AMS and HACE

The Lake Louise Scoring (LLS) system was designed to evaluate adults for symptoms of AMS. Symptoms include headache, gastrointestinal symptoms, fatigue and/or weakness, dizziness/lightheadedness and difficulty sleeping (Roach *et al.* 1993). If present, each symptom was rated according to LLS from 0 to 3. The diagnosis of AMS was made if two items were present and LLS ⩾3 (Roach *et al.* 1993). We used a modified version of the LLS in which, contrary to the conventional scoring criteria, headache was not used as a compulsory symptom since its presence or absence was not consistently reported in the text. This is in line with John West who stated that ‘the basic Lake Louise procedure could be used without the requirement that headache be essential’ (West, 2011). According to LLS, the diagnosis of HACE was made in persons with AMS and at least one of the following symptoms of mental status change: lethargy/lassitude, disorientation/confusion, stupor/semi-consciousness (Roach *et al.* 1993). Additional somatic conditions such as breathing problems, frostbite, snow blindness, starvation and use of supplementary oxygen were recorded (YES/NO/NA).

### Assessment of accidents

It was scored whether there was an accident or near accident (YES/NO/NA). Accidents which occurred fatefully, such as avalanches or collapses of seracs, were not recorded.

### Statistical analysis

To identify similar groups and to assign them to diagnoses, we performed a cluster analysis using the SPSS^®^ Two Step clustering method (SPSS Inc., 2007). In order to reveal natural clusters within the dataset, the most important factors from a theoretical point of view were identified. This included all symptoms listed in the CRDPSS, anxiety, tour-related factors and somatic conditions. Firstly, the clustering procedure was performed on the whole list of factors, and for the final phase, we chose the factors with the value of importance higher than 0.4 (see Table 1 for details).

**Table 1.** Distribution of episodes in the relevant clusters

	Cluster 1	Cluster 2	Cluster 3
Cluster characteristics			
Name of cluster	‘No psychosis’	‘Psychosis plus’	‘Isolated psychosis’
Abbreviated name of cluster	PSY^NO^	PSY^PLUS^	PSY^ISO^
Per cent and (absolute number) of episodes classified in the cluster	51% (42/83)	22% (18/83)	28% (23/83)
Factors for clustering			
Primary symptoms of psychosis (importance = 1)	No (98%)	Yes (100%)	Yes (100%)
Psychosis (importance = 0.96)	No (100%)	Yes (100%)	Yes (91%)
Mental status change (importance = 0.6)	No (74%)	Yes (100%)	No (100%)
HACE (importance = 0.42)	No (86%)	Yes (72%)	No (100%)
Third person phenomenon (importance = 0.41)	No (100%)	No (56%)	Yes (61%)

The factors are ordered according to their importance in the clustering procedure. When clinical expert knowledge was used to classify the individual episodes a very good concordance with the clustering algorithm was achieved classifying only 2% (2/83) of episodes in a divergent cluster. Cluster analysis was done using the SPSS Two Step clustering method

Nominal variables were analysed using χ^2^ test and *z*-test to compare column proportions. Continuous variables were analysed using Kruskal–Wallis test. Odds ratio (OR) was calculated to evaluate the association of disease with accidents or near accidents. *P* < 0.05 was considered significant in all analyses.

## Results

### Identification of three distinct symptom clusters

**Fig. 1.**
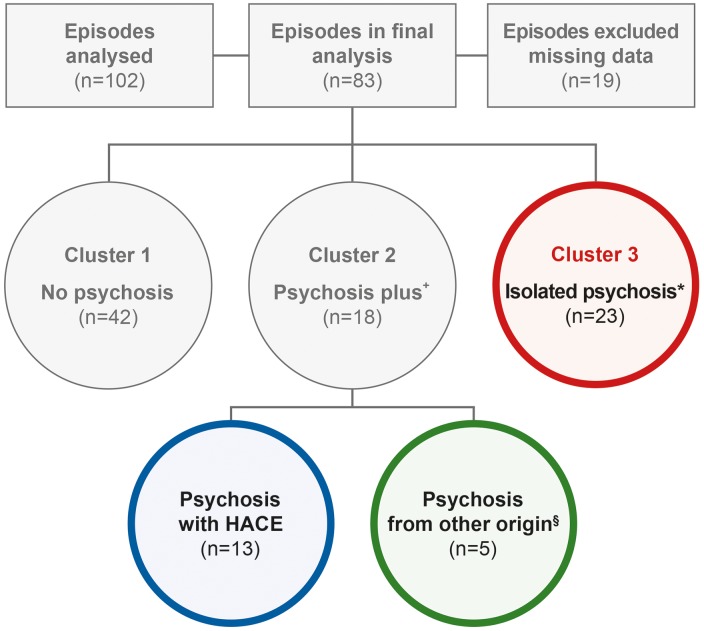
Diagram displaying the relationship of psychosis, mental status change and HACE. Overall 102 episodes were analysed (absolute numbers in brackets) of which 83 episodes were included in the final analysis. + With mental status change; * without mental status change; § psychosis from other origin includes cases with mental status change due to, e.g. infection, dehydration or drugs. HACE: high-altitude cerebral oedema.

Data were collected from 60 subjects describing 102 episodes. Using cluster analysis, we identified three distinct clusters of symptoms in 83 episodes, in which complete information on all clustering variables was available (Table 1). Cluster 1 included episodes without psychosis ‘no psychosis’ (PSY^NO^), cluster 2 episodes with psychosis plus symptoms of mental status change ‘psychosis plus’ (PSY^PLUS^) and cluster 3 episodes with psychosis without symptoms of HACE or mental status change from other origins ‘isolated psychosis’ (PSY^ISO^). In the PSY^PLUS^ cluster, psychosis co-occurred with HACE in 16% (13/83) of episodes, while in 6% (5/83) of episodes, it occurred together with mental status change from other origins (Fig. 1).

### Subject and mountain-related data

**Fig. 2.**
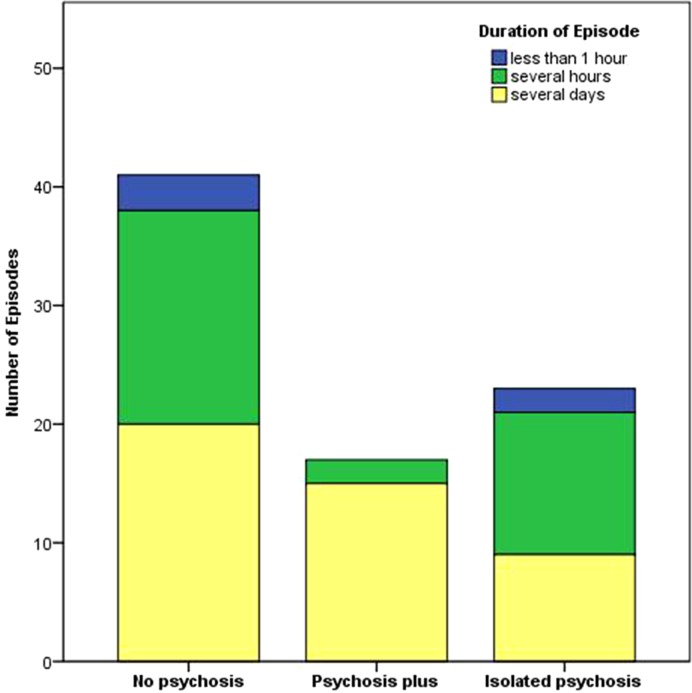
Bar chart depicting the duration of the episodes in the three clusters. The duration differed between the three clusters (χ^2^ test, *p* = 0.031), with episodes in the PSY^PLUS^ cluster lasting longer than those in the PSY^NO^ and PSY^ISO^ clusters (*z*-test < 0.05 for PSY^NO a^, PSY^PLUS b^, PSY^ISO a^). For *z*-test, each superscript letter denotes a subset of categories whose column proportions do not differ significantly from each other at the 0.05 level.

The mean number of episodes per subject was 1.9 (range 1–8), 82% (68/83) of the episodes occurred in males and 18% (15/83) in females; with no difference between the three clusters (χ^2^ test, *p* = 0.296). The mean age of subjects was 34.2 ±  8.4 (mean ± s.d.) years (Kruskal–Wallis test, *p* = 0.315). The altitude at which episodes occurred was 7280 m ± 1293 (mean ± s.d.; Kruskal–Wallis test, *p* = 0.932). The duration of episodes differed between the three clusters (χ^2^ test, *p* = 0.031), with episodes in the PSY^PLUS^ cluster lasting longer than those in the PSY^NO^ and PSY^ISO^ clusters (*z*-test < 0.05 for PSY^NO a^, PSY^PLUS b^, PSY^ISO a^; Fig. 2). Psychotic episodes were fully reversible. Most of the episodes occurred in the Himalayas [55% (46/83)] and Karakorum [34% (28/83); Supplemental Material 2].

### Frequency of HACE and AMS

AMS occurred in 48% (40/83) of episodes and the frequency did not differ significantly between the three clusters (χ^2^ test, *p* = 0.053). HACE occurred in 23% (19/83) of episodes: in 10% (6/42) of episodes in the PSY^NO^ cluster, in 72% (13/18) of episodes in the PSY^PLUS^ cluster and in 0% (0/23) of episodes with isolated psychosis (χ^2^ test, *p* = 0.000, *z*-test < 0.05 for PSY^NO a^, PSY^PLUS b^, PSY^ISO a^).

### Analysis of symptoms of psychosis across the different symptom clusters

We analysed the distribution of different symptoms of psychosis across the three clusters (Table 2). Overall, hallucinations were found in 42% (35/83) and perceptional disturbances (hallucinations, depersonalisation and illusions) in 58% (48/83) of episodes. Perceptional disturbances were perceived as neutral in 60% (29/48), as helpful or comforting in 23% (11/48) and as frightening or dangerous in 17% (8/48).

**Table 2.** Frequency of symptoms across the different clusters

	PSY^NO^	PSY^PLUS^	PSY^ISO^	Missing	*p*	*z*-test
Perceptional disturbances	29% (12/42)	89% (16/18)	87% (20/23)	0	0.000	PSY^NO a^ PSY^PLUS b^ PSY^ISO b^
Hallucinations all modalities	2% (1/42)	83% (15/18)	83% (19/23)	0	0.000	PSY^NO a^ PSY^PLUS b^ PSY^ISO b^
Hallucinations visual	0% (0/42)	61% (11/18)	30% (7/23)	0	0.000	PSY^NO a^ PSY^PLUS c^ PSY^ISO b^
Hallucinations acoustic	2% (1/42)	61% (11/18)	22% (5/23)	0	0.000	PSY^NO a^ PSY^PLUS c^ PSY^ISO b^
Hallucinations somaesthetic	0% (0/42)	17% (3/18)	13% (3/23)	0	0.033	PSY^NO a^ PSY^PLUS b^ PSY^ISO b^
Hallucinations olfactory	0% (0/42)	0% (0/18)	0% (0/23)	0	–	–
Hallucinations gustatory	0% (0/42)	0% (0/18)	0% (0/23)	0	–	–
Third person phenomenon	0% (0/42)	44% (8/18)	61% (14/23)	0	0.000	PSY^NO a^ PSY^PLUS b^ PSY^ISO b^
Illusion	12% (5/42)	11% (2/18)	26% (6/23)	0	0.269	–
Depersonalisation	10% (4/42)	22% (4/18)	33% (3/23)	0	0.413	–
Delusion	0% (0/41)	72% (13/18)	44% (10/23)	1	0.000	PSY^NO a^ PSY^PLUS b^ PSY^ISO b^
Disorganised speech	0% (0/42)	24% (4/17)	91% (2/22)	3	0.008	PSY^NO a^ PSY^PLUS b^ PSY^ISO b^
Abnormal psychomotor behaviour	36% (15/42)	78% (14/18)	23% (5/22)	1	0.001	PSY^NO a^ PSY^PLUS b^ PSY^ISO a^
Decrease	31% (13/42)	79% (14/18)	18% (4/22)	1	0.000	PSY^NO a^ PSY^PLUS b^ PSY^ISO a^
Increase	7% (3/42)	17% (3/18)	5% (1/22)	1	0.354	–
Negative symptoms	52% (22/42)	88% (15/17)	38% (8/21)	3	0.006	PSY^NO a^ PSY^PLUS b^ PSY^ISO a^
Decrease in motivation	19% (8/42)	71% (12/17)	10% (2/21)	3	0.000	PSY^NO a^ PSY^PLUS b^ PSY^ISO a^
Decrease in communication	31% (13/42)	59% (10/17)	10% (2/21)	3	0.005	PSY^NO a^ PSY^PLUS b^ PSY^ISO a^
Decrease in affective expression	41% (17/42)	71% (12/17)	29% (6/21)	3	0.028	PSY^NO a^ PSY^PLUS b^ PSY^ISO a^
Impaired cognition	62% (26/42)	100% (18/18)	86% (19/22)	1	0.003	PSY^NO a^ PSY^PLUS b^ PSY^ISO b^
Accelerated	0% (0/42)	0% (0/18)	0% (0/22)	1	–	–
Slowed	31% (13/42)	39% (7/18)	18% (4/22)	1	0.338	–
Automated	36% (15/42)	89% (16/18)	68% (15/22)	1	0.000	PSY^NO a^ PSY^PLUS b^ PSY^ISO b^
Disorganised	36% (15/42)	89% (16/18)	68% (15/22)	1	0.000	PSY^NO a^ PSY^PLUS b^ PSY^ISO b^
Depression	31% (13/42)	53% (9/17)	19% (4/21)	3	0.081	–
Mania	50% (21/42)	26.7% (4/15)	33.3% (7/21)	5	0.203	–
Anxiety	36% (15/42)	53% (8/15)	36% (8/22)	4	0.462	–

Items of the Clinician-Rated Dimensions of Psychosis Symptom Severity Scale are shaded in grey. The χ^2^ test was used to analyse PSY^NO^ *v.* PSY^PLUS^ *v.* PSY^ISO^. *P* < 0.05 was considered significant in all analyses. For *z*-test each superscript letter denotes a subset of categories whose column proportions do not differ significantly from each other at the 0.05 level. Abbreviations: PSY^NO^: cluster 1 without psychosis; PSY^PLUS^: cluster 2 with psychosis in the context of HACE or mental status change from other origins; PSY^ISO^: cluster 3 with isolated psychosis.

### Risk factors and characteristics of episodes with psychosis

The distribution of potential risk factors of psychosis and accompanying somatic symptoms across the three clusters was analysed (Table 3).

**Table 3.** Analysis of risk factors of psychosis and associated somatic conditions

	PSY^NO^	PSY^PLUS^	PSY^ISO^		*p*1		*p*2
	Cluster 1	Cluster 2	Cluster 3	Missing	Cluster 1 *v.* 2 *v.* 3	*z*-test	Cluster 1 *v.* 2 + 3
Risk factors							
Ascent	53% (16/30)	43% (6/14)	53% (10/19)	20	0.796	–	0.701
Danger	26% (11/42)	56% (10/18)	26% (6/23)	0	0.062	–	0.212
Alone	21% (9/42)	39% (7/18)	35% (8/23)	0	0.301	–	0.128
Self-report	91% (38/42)	72% (13/18)	87% (20/23)	0	0.179	–	0.196
Associated somatic conditions							
Snow blindness	13% (5/40)	20% (3/15)	17% (4/23)	5	0.751	–	0.469
Breathing problems	38% (16/42)	38% (6/16)	44% (10/23)	2	0.899	–	0.787
Starvation	10% (4/40)	53% (8/15)	17% (4/23)	5	0.002	PSY^NO a^ PSY^PLUS b^ PSY^ISO a^	0.018
Frostbite	48% (19/40)	87% (13/15)	48% (11/23)	5	0.024	PSY^NO a^ PSY^PLUS b^ PSY^ISO a^	0.165
Supplemental oxygen	5% (2/41)	28% (5/18)	13% (3/23)	1	0.046	PSY^NO a^ PSY^PLUS b^ PSY^ISO a, b^	0.043

χ^2^ test (*p*1) was used to analyse PSY^NO^ *v.* PSY^PLUS^ *v.* PSY^ISO^ and a second χ^2^ test (*p*2) was used to analyse cases without psychosis (cluster 1) *v.* cases with psychosis (clusters 2 + 3). *P* < 0.05 was considered significant in all analyses. For the *z*-test, each superscript letter denotes a subset of categories whose column proportions do not differ significantly from each other at the 0.05 level. Abbreviations: PSY^NO^: cluster without psychosis; PSY^PLUS^: cluster with psychosis in the context of HACE or mental status from other origins; PSY^ISO^: cluster with isolated psychosis.

### The association of psychosis with near accidents or accidents

We analysed the distribution of accidents and near accidents across the clusters: 12% (5/42) of cases were associated with accidents or near accidents in the PSY^NO^ cluster, 59% (10/17, one missing) in PSY^PLUS^ and 22% (5/23) in PSY^ISO^ (χ^2^ test, *p* = 0.001, *z*-test < 0.05 for PSY^NO a^, PSY^PLUS b^, PSY^ISO a^). Overall, episodes without psychosis (cluster 1) were compared with episodes with psychosis (clusters 2 + 3). The latter were associated with a higher number of near accidents and accidents [cluster 1 12% (5/42), clusters 2 + 3 38% (15/40, one missing), χ^2^ test, *p* = 0.007, OR 4.44]. Episodes where individuals were considered to display symptomology congruent with AMS were not associated with a higher number of accidents or near accidents compared with episodes without AMS [28% (11/40) *v.* 21% (9/42, one missing), χ^2^ test, *p* = 0.522, OR 1.28]. While episodes where individuals were considered to display symptomology congruent with HACE showed higher number of accidents and near accidents compared with episodes without HACE [42% (8/19) *v.* 19% (12/63, one missing), χ^2^ test, *p* = 0.04, OR 3.09].

## Discussion

The major findings of the present study are: (1) psychosis occurs during exposure to HA; (2) it can occur in isolation, or together with HACE or mental status change of other origins (e.g. infection, dehydration); (3) isolated HA psychosis should thus be considered as a separate HA-related disease (Fig. 1); (4) HA psychosis is associated with an increased rate of near accidents and accidents.

The incidence of psychosis was not formally assessed in previous studies, but the occurrence of hallucinations, one of the core features, was very variable in the literature: Wu *et al.* (2006) reported hallucinations in 3% of cases with HACE, while Wilson *et al.* (2009) reported them in 32% of climbers above 7500 m and Brugger *et al.* (1999) found hallucinatory experiences in seven of eight (88%) world-class climbers who reached altitudes above 8500 m without supplementary oxygen. In the present study, hallucinations occurred in 42% (35/83) of episodes at a mean altitude of 7280 m; of these episodes in 34% (12/35), the hallucinations occurred together with HACE. Considering the severity and high mortality of HACE, it is most likely that hallucinations and/or psychosis can also occur outside of HACE. In many of our rated episodes, plus in the personal account of one of the co-authors (I.T. Supplemental Material 3), and also in the study by Brugger *et al*., alpinists with symptoms of psychosis descended unaided from the mountain and showed no permanent neurological sequelae (Brugger *et al.* 1999); thus, it is unlikely that they suffered from HACE. Isolated HA psychosis could be related to the syndrome of transient HA neurological dysfunction, which has been previously proposed as a separate complication at HA, distinct from HACE (Firth & Bolay, 2004).

It has long been recognised that signs and symptoms of psychosis are on a continuum with normal mental states (Allardyce *et al.* 2007), and that there is an individual susceptibility towards hallucinations and psychosis also in healthy individuals (Morrison *et al.* 2002). Possible mechanisms behind HA-related psychosis could be social and sensory deprivation in conjunction with psychological stress (Daniel & Mason, 2015), as well as dysfunction of the temporoparietal junction and angular gyrus due to hypoxia, hypoglycaemia and cold (Lempert *et al.* 1994; Feddersen *et al.* 2007). Imaging alterations in HACE include the white matter particularly in the splenium of the corpus callosum (Hackett & Roach, 2004), which has been implicated in the development of psychosis in patients at risk of schizophrenia (von Hohenberg *et al.* 2014). Research in animal models has shown that moderate hypoxia (12.5% oxygen) increases dopamine (79%) and serotonin (26%) in the extracellular fluid of the rat striatum and both neurotransmitters remain elevated even after the initial reintroduction of normoxic air (Broderick & Gibson, 1989). Both dopamine and serotonin metabolism are linked to hallucinations (Rolland *et al.* 2014), and there is evidence of altered availability of these neurotransmitters in psychosis, also in the context of schizophrenia (Geyer & Vollenweider, 2008; Howes & Kapur, 2009).

While perceptional disturbances were perceived as neutral or helpful/comforting by the majority of afflicted subjects in the current study, such psychiatric symptoms can also be detrimental as the association of psychosis with accidents and near accidents showed. This is in line with a previous study, showing that cognitive disturbances (no exact specification of symptoms) may predict death on Mount Everest (Firth *et al.* 2008). Though accidents due to objective dangers such as avalanches or serac collapses were not recorded in the current study, in some cases, their inclusion could have been justified owing to poor route selection or climbing in unsuitable conditions due to psychosis-induced errors of judgement.

The incidence of HACE is mostly reported to be about 0.1–2% at altitudes between 3500 and 5500 m (Hochstrasser *et al.* 1986; Hackett *et al.* 1998; Wu *et al.* 2006), but in situations of fast ascent numbers as high as 31% have been found (Basnyat *et al.* 2000). In our study, HACE was present in 23% of episodes. The reported differences in HACE incidence in the literature are probably due to heterogeneous populations, in addition to possible differences in diagnostic criteria or differences in evaluated altitudes. The mean altitude in the present study was above 7000 m, while other studies examined much lower altitudes (Basnyat *et al.* 2000; Hackett & Roach, 2004; Wu *et al.* 2006). The most important aspect for diverging incidences seems to be that AMS and HACE are two extremes of a continuum, and that the cut-off point where severe ‘AMS’ symptomology is called ‘HACE’ is not always easy to determine. HACE could be underdiagnosed in the field as mountaineers may ignore minor alterations of the mental status. In some cases, the diagnosis will be made only once mental status is severely impaired or coma occurs (Wu *et al.* 2006), which is considered the final stage of HACE before death (Hackett & Roach, 2004). This could also be due to the nature of the diagnostic criteria of mental status change used for the diagnosis of HACE. While ‘coma’ is relatively easy to diagnose, the judgement on ‘lethargy’ will differ between evaluators: it would be clearer if the quantitative changes in consciousness were rated, e.g. according to the Glascow Coma Scale. ‘Psychological changes’, including emotional changes or irrational behaviour (27%), and disturbances in consciousness (79%), are common in HACE and are considered indicative of organic brain dysfunction (Ryn, 1988; Wu *et al.* 2006) (classified as delirium in DSM-5). HA-related organic brain dysfunction (delirium), with or without psychosis, can also occur without HACE (Ryn, 1988; Basnyat, 2002). Such cases were also identified in the current study (Fig. 1). Possible underlying pathogenetic mechanisms are hypothermia, infection, medication or dehydration; however, these aspects have not been investigated in the current study (Pendlebury *et al.* 2015). Additional psychiatric symptoms such as mania, depression or anxiety were also assessed in the current study and showed elevated prevalence at HA: a finding in line with previous reports (Ryn, 1988; Shukitt-Hale *et al.* 1991; de Aquino Lemos *et al.* 2012).

There are several limitations to the present study. The most evident limitation is the method of data collection; inter-rater *κ*s were, however, very consistent. This was mostly attributable to the training of the raters. The way in which the literature search was conducted (i.e. the use of German key words in the German site of an online book shop) is a potential limitation; it would also be interesting to include the American book market. Within this study, we assume that there is an inherent bias because episodes with psychosis are more likely to be reported. Additionally, due to the inclusion of retrospective accounts within this study, there also exists a bias for survival, since only people surviving the climb can later report their symptoms. We addressed this question by analysing whether there was a difference in self-report and observed episodes.

In conclusion, psychosis can occur at very high and extreme altitude, and reportedly in the absence of other signs and symptoms of HACE. Isolated HA psychosis should thus be considered a distinct and separate HA-related syndrome. It is important to inform subjects who intend to go to HA about the possibility of psychosis, in addition to the well-recognised somatic HA disorders. This should be part of the information campaign for HA travellers. Cognitive strategies (e.g. reality testing, Smailes *et al.* 2015) should be considered and practiced beforehand. This information has the potential to increase safety at HA, especially when with a partner or in a group. These findings are not only important for climbers and mountaineers but also for occupational HA work. To better describe HA psychosis, to determine its incidence, risk and trigger factors, treatment and patient's outcome, a prospective observational study should be performed. Additionally, *in vitro* studies could further ameliorate knowledge of the underlying pathophysiology. Since isolated psychosis at altitude shows some clinical features similar to schizophrenia, it could potentially serve as a reversible model of the disease; thus aiding in the investigation of pathophysiological concepts or new treatments for schizophrenia and related disorders.

## Supplementary material

For supplementary material accompanying this paper visit http://dx.doi.org/10.1017/S0033291717003397. click here to view supplementary material
