# Supplementary material for: Isolated psychosis during exposure to very high and extreme altitude – characterisation of a new medical entity
Source: Psychol Med. 2017 Dec 5;48(11):1872–9. doi: 10.1017/S0033291717003397 (PMC6088769; doi:10.1017/S0033291717003397)
Supplement: Supplementary file 1 [file S0033291717003397sup001.docx]

**Supplemental Material**

**Supplemental material 1 - analysed primary literature**


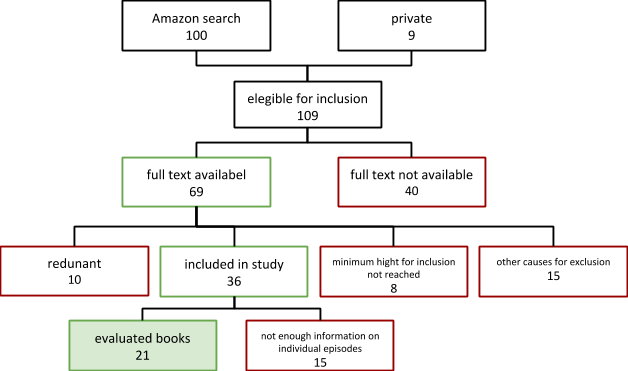


Figure 1. Flow chart of the process of episode identification. The numbers indicate the absolute number of books evaluated.

The study was done using the German edition of all listed literature; we list here the equivalent English titles, where available. For some titles we did not find an English equivalent.

- Auen, Klaus M. Dem Himmel nah, Schweinfurt: Wiesenburg 2014 (German Edition only)
- Bowley, Graham. No Way Down: Life and Death on K2, London: Penguin 2011
- Buhl, Hermann. Nanga Parbat Pilgrimage: The Lonley Challenge, Leicesteshire: Baton Wicks 1998
- Rose, David and Douglas, Ed. Regions of the Heart: The Triumph and Tragedy of Alison Hargreaves, Washington DC: National Geographic 2001
- Diemberger, Kurt. The Endless Knot: K2, Mountain of Dreams and Destiny, Seattle: Mountaineers Books 1991
- Gammelgaard, Lene. Climbing High: A Woman's Account of Surviving the Everest Tragedy, New York: Harper Paperbacks 2000
- Hall, Lincoln. Dead Lucky: Life After Death on Mount Everest, New York: Tarcher 2009
- Harrer, Heinrich. The White Spider, New York: Tarcher 1998
- House, Steve. Beyond the Mountain, Ventura: Patagonia 2012
- Kaltenbrunner, Gerlinde. Mountains in my Heart. A Passion for Climbing, Seattle: Mountaineers Books, 2014
- Krakauer, Jon. Into Thin Air: A Personal Account of the Mt. Everest Disaster, New York: Anchor 1999
- Kropp, Göran. Ultimate High: My Everest Odyssey, Ludlow: Discovery Books 1999
- McDonald, Bernadette. Freedom Climbers, Sheffield: Vertebrate Graphics, 2012
- Mees, Klaus. Grenzerfahrungen in der Todeszone: Höhenbergsteigen hautnah erzählt, München: Bruckmann 2013 (German Edition only)
- Messner, Reinhold. To the Top of the World: Alpine Challenges in the Himalaya and Karakoram, Seattle: Mountaineers Books 1999
- Messner, Reinhold. The Naked Mountain, Ramsbury: The Crowood Press Ltd 2005
- Oelz, Oswald, Mit Eispickel und Stethoskop, München: Malik 2013 (German Edition only)
- Saler, Hans, [Zwischen Licht und Schatten: Die Messner-Tragödie am Nanga Parbat](http://www.amazon.de/Zwischen-Licht-Schatten-Messner-Tragödie-Parbat/dp/3927743658/ref=sr_1_58?s=books&ie=UTF8&qid=1406578322&sr=1-58&keywords=bergsteigen), München: A1 Verlag, 2003 (German Edition only)
- Simpson, Joe. This Game of Ghosts, London: Jonathan Cape 1993
- Simpson, Joe. Touching the Void, New York: Vintage 1998
- Twight, Mark. Kiss or Kill: Confession of a Serial Climber, Seattle: Mountaineers Books, 2002

**Supplemental material 2 - List of the mountains where the episodes occurred**

| **Mountain range** | **Mountain** | **Height in meters** | **Number of episodes** |
| --- | --- | --- | --- |
| Himalayas | Mt.Everest  Nanga Parbat  Annapurna  Tarke Kang (Glacier Dome)  Kangchendzönga  Nupstse Shar I (Nupstse East)  Manaslu  Makalu  Dhaulagiri  Pachermo  Lhotse | 8.848  8.125  8.091  7.193  8.586  7.804  8.163  8.481  8.167  6.187  8.516 | 18  13  1  1  3  2  3  1  2  1  1 |
| Karakorum | K2  Broad Peak  Chogolisa  Hidden Peak (Gasherbrum I)  Gasherbrum II | 8.611  8.051  7.665  8.080  8.034 | 13  5  3  2  5 |
| European Alps | Eiger  Grandes Jorasses | 3.970  4.208 | 3  1 |
| Alaska Range | Denali (Mt. McKinley) | 6.164 | 1 |
| Andes | Siula Grande | 6.344 | 3 |
| Caucasus Mountains | Mt. Elbrus | 5.642 | 1 |

**Supplemental material 3- high altitude psychosis, a personal experience of Iztok Tomazin**

Daulagiri winter alpine style ascent December 1987

I climbed the summit in four days as a member (and doctor) of small team of Slovenian climbers. I had almost no acclimatization but a lot of motivation. During the acclimatization in the mountains near Daulagiri I spent only 1.5 hours at 6000 meters and two days above 5000m. Then I had to descend because of severe gastroenteritis (giardiasis).


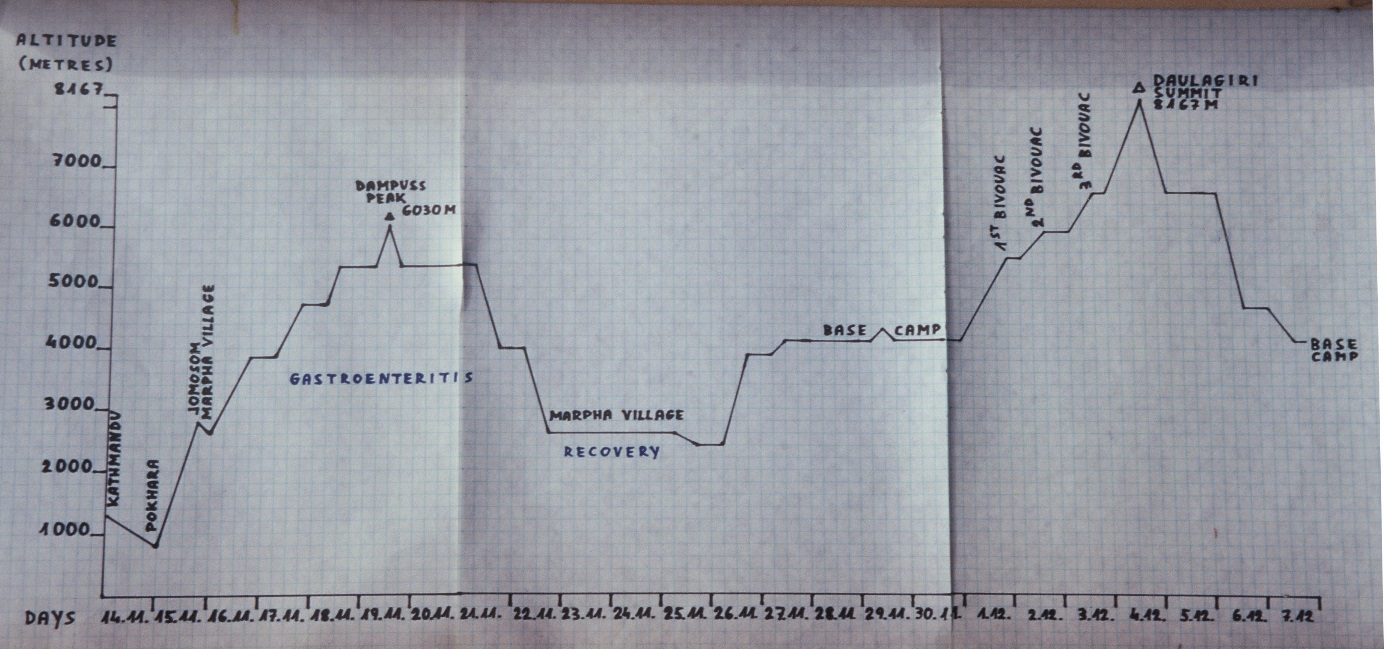


On the summit day we started from our last bivouac at 6600m climbing simultaneously. Two friends (Stane and Pavle) turned back at around 8000 m and descend because of altitude problems, but my friend Marjan and I continued towards the summit. I reached the summit after 17 hours of continuous climbing. I was vomiting and extremely exhausted. The heavy backpacks (about 25 kg), the difficult climbing on the east face of the mountain and the lack of acclimatization all had influences on my exhaustion. Marjan came to the summit 20 minutes after myself. I waited for him on the summit in extremly low temperatures and very strong winds. If he would not have come, I would probably have stayed on the summit and frozen to death, because I had the idea fixed in my mind that I must wait for my friend at the summit. We then started to descend together. Very soon it became dark, we got separated (we don’t remember how it happened) and I continued to descend alone in the dark. We didn’t see each other during the rest of the descent. Soon after we separated I started to have hallucinations. They were mostly very vivid feelings that some people were descending together with me (“mountain guides”). I was arguing with them because they pressured me with some advice which I didn’t want to follow. The most crucial point came when I missed the right way (the only possible passage) from the summit slopes to the northwest rib where the descending route continues. I came to the top of the east face and suddenly a 2000 meter vertical drop was below my feet. I was in great distress, very exhausted, dehydrated and hypothermic and trapped in difficult terrain without knowledge where to descend and with great wish/need to find a shelter where I could rest and sleep and be safe and warm. But I was still at an altitude of almost 8000 meters. Suddenly the hallucinated “mountain guides” started to talk with me with very sweet and energetic advice: “jump down the east face and in few seconds you will be on a flat, safe place 2000 metres lower, this will solve all your problems”. So I was standing there, at the very edge of the east face, prepared to jump because these voices almost convinced me that jumping down the face is the best or only solution to my problems. I almost jumped and this would meant death with a 100% chance. At the last moment, just before I jumped, a quick thought came to my mind: “what if this is not true? Then I will die”. So I made the decision to take a test - I jumped only approximately 2 metres to a small ledge. I experienced severe pain when I hit with the rock on the ledge with my legs in boots and crampons. Through the pain I immediately and very clearly recognized the situation and what would happen if I would jump down the face. So I continued to struggle down climbing the edge of the face and finally I found the right way down and continued descending. With coming lower, hallucinations slowly disappear. I reach our bivouac at 6500 meters after several hours of climbing, without hallucinations but still with cognitive impairment: I saw our tent but I had no reflection, no happiness no feelings about it. I should be very happy to finally reach the safe shelter, where I can rest and sleep and eat. But my only thought was: “I see the tent”… and then I continued to descend! Luckily our friend Stane, who was already at the bivouac tent, heard my steps and when he recognized I didn’t stop he started to shout and made me stop the descend. Without his intervention I would continue the descent, because I was so determined to descend. I have no idea when I would have stopped – probably at the stage of complete exhaustion or I would have fallen in one of many crevasses on the glacier lower down.

At the same time my friend Marjan was descending from the summit. We didn’t know how far we were one from another. I remember that I saw a man descending few hundred meters below me, but my hypoxic, exhausted brain didn’t make the conclusion, that this was my friend, and that we should descend together to improve our chances and safety. My only thought was: a man, without any reflexion. Marjan was also exhausted and had many technical difficulties during the descend. He was very tired and sleepy. During his descent he also had frequent hallucinations: our friend Stane, (who had turned back at 8000 meters several hours ago and was already at the bivouac) was descending near him and all the time he suggested to him kindly:”Marjan, you should lay down and rest, sleep and everything will be fine”. If Marjan would have listened to him he would have newer woken up, because it was more than minus 40 degrees and he was extremely exhausted. Fortunately he didn’t listen to the hallucinations of his friend and continued with his descent. He reached the bivouac at 6500 m a few hours after me.
